# Separation and Purification of Hydroxyl-α-Sanshool from *Zanthoxylum armatum* DC. by Silica Gel Column Chromatography

**DOI:** 10.3390/ijms24043156

**Published:** 2023-02-05

**Authors:** Jinxi Cheng, Xiaoyan Hou, Qiang Cui, Guanghui Shen, Shanshan Li, Qingying Luo, Man Zhou, Hong Chen, Zhiqing Zhang

**Affiliations:** 1 College of Food Science, Sichuan Agricultural University, Ya’an 625014, China; 2 Deyang Food & Drug Safety Inspection and Testing Center, Deyang 618029, China

**Keywords:** *Zanthoxylum armatum* DC., hydroxyl-α-sanshool, separation, silica gel column chromatography

## Abstract

Hydroxyl-α-sanshool is the main alkylamide produced by *Zanthoxylum armatum* DC., and it is responsible for numbness after consuming *Z. armatum*-flavored dishes or food products. The present study deals with the isolation, enrichment, and purification of hydroxyl-α-sanshool. The results indicated that the powder of *Z. armatum* was extracted with 70% ethanol and then filtrated; the supernatant was concentrated to get pasty residue. Petroleum ether (60–90 °C) and ethyl acetate at a 3:2 ratio, with an Rf value of 0.23, were chosen as the eluent. Petroleum ether extract (PEE) and ethyl acetate–petroleum ether extract (E-PEE) were used as the suitable enriched method. Afterward, the PEE and E-PEE were loaded onto silica gel for silica gel column chromatography. Preliminary identification was carried out by TLC and UV. The fractions containing mainly hydroxyl-α-sanshool were pooled and dried by rotary evaporation. Lastly, all of the samples were determined by HPLC. The yield and recovery rates of hydroxyl-α-sanshool in the p-E-PEE were 12.42% and 121.65%, respectively, and the purity was 98.34%. Additionally, compared with E-PEE, the purity of hydroxyl-α-sanshool in the purification of E-PEE (p-E-PEE) increased by 88.30%. In summary, this study provides a simple, rapid, economical, and effective approach to the separation of high-purity hydroxyl-α-sanshool.

## 1. Introduction

Green Sichuan pepper (*Zanthoxylum armatum* DC.), known as “Qinghuajiao” in Chinese, is a popular perfume species of two main varieties in southwest China [1]; it belongs to the genus *Zanthoxylum* of the Rutaceae family. Hydroxyl-α-sanshool was first isolated from the fresh bark and pericarps of Japanese *Zanthoxylum* species in 1982 by Yasuda et al. [2,3]. It has also been shown to be present in the young leaves [4], flowers, and seeds [5] of *Zanthoxylum*, one of the predominant classes of long-chained polyunsaturated amides occurring in the *Zanthoxylum* species, responsible for numbness after consuming *Z. armatum*-flavored dishes or food products. Researchers who have analyzed more than 30 types of unsaturated fatty acid amides (alkylamines) from *Zanthoxylum* species have found essential pungent and tingling compounds, which include α-, β-, γ-, and δ-sanshool and their derivatives [6]. Among these compounds, hydroxyl-α-sanshool greatly contributes to numbness from *Z. armatum* upon consumption [7]. The contents of hydroxyl-α-sanshool, hydroxyl-β-sanshool, and hydroxyl-γ-sanshool were 15.69%, 4.76%, and 0.96%, which were high compared to all other alkylamines in this *Zanthoxylum* species, respectively [6]. In addition, recent reports have demonstrated that amides in *Zanthoxylum* offer a series of physiological activities in curing inflammatory diseases [8,9], killing insects [10], relieving pain [11], etc. Therefore, as the most abundant amides component in *Z. armatum*, hydroxyl-α-sanshool is of particular interest since it could serve as a quality indicator of commercial *Z. armatum* products in edible and medicinal products [12]. Based on its popular uses, including food processing and pharmacological activities, there is great demand for large quantities of high-purity hydroxyl-α-sanshool. Thus, there is a research focus on exploring separation and purification approaches for extracting it.

The separation and purification of hydroxyl-α-sanshool can be challenging due to its three conjugated double bonds, which are sensitive to oxygen [13] and easily undergo isomerization, hydrolysis, and oxidation [14]. Several strategies have been reported for the separation of amides from the different parts of *Zanthoxylum* using various methodologies. Among them, preparative high-performance liquid chromatography (Prep-HPLC) and semi-preparative HPLC are commonly used for the separation of hydroxyl-α-sanshool [3,12,15,16]. This separation method has obvious advantages in producing high-purity hydroxyl-α-sanshool. However, it is accompanied by the problem of tedious sample preparation and diseconomy. Although high-speed counter-current chromatography (HSCCC) [17,18] has a high recovery rate and milligram quantity and is easy to operate for the separation of hydroxyl-α-sanshool, the main disadvantages of this method are that it is time-consuming, has low resolution, and requires large volumes of solvents. Furthermore, amides isolated from *Zanthoxylum* by supercritical CO_2_ are in high yield [19]. This method is limited by the high capital requirement for the instruments, which may not be a viable economic strategy, and a low purification rate. In another report, molecularly imprinted polymers were proposed to prepare a molecularly imprinted solid-phase extraction column for the separation of acid amide components from *Zanthoxylum* oils [20], but preparing the molecularly imprinted polymers might be cumbersome. Overall, the limitations of the reported methods mainly include high capital input, high time consumption, and tedious preparation. Hence, these limitations warrant the exploration of a simple, economical, and efficient method for the separation and purification of hydroxyl-α-sanshool from *Z. armatum*.

Silica gel is the most common adsorbent in chemical analysis. Silica gel chromatography has been widely used in separation and purification due to its various advantages, which are low energy consumption, high separation purity, simple operation without large-scale equipment, etc. At present, due to the different mechanisms of substance separation, the main separation types of column chromatography are: ion exchange chromatography [21,22], gel column chromatography [23,24], adsorption chromatography [25,26], isoelectric focusing chromatography [27,28,29], etc. Among them, ion exchange chromatography is commonly used to separate sugars [30,31], enzymes [32], proteins [33], amino acids [34,35], etc. Gel column chromatography is widely used in the purification of macromolecular substances such as enzymes [36], polysaccharides [37], and proteins [38], while adsorption chromatography is mainly used in the separation of alkaloids [39], pigments [40,41] and other small molecular substances. In this work, based on the characteristics of the separated target substance, hydroxyl-α-sanshool belongs to a kind of small molecule substance. Adsorption chromatography is a chromatographic separation and analysis method based on the different adsorptivities of different compounds [25]. Therefore, adsorption column chromatography technology has been selected to separate the hydroxyl-α-sanshool from *Z. armatum.*

Considering the need for an economically viable method to separate and purify a large quantity of hydroxyl-α-sanshool, this study was conducted to develop a rapid economical method by silica gel chromatography for the separation of hydroxyl-α-sanshool from *Z. armatum* and to confirm its identity by spectral analysis using UV, TLC, and HPLC.

## 2. Results and Discussion

### 2.1. Selection of Eluent

**Figure 1:**
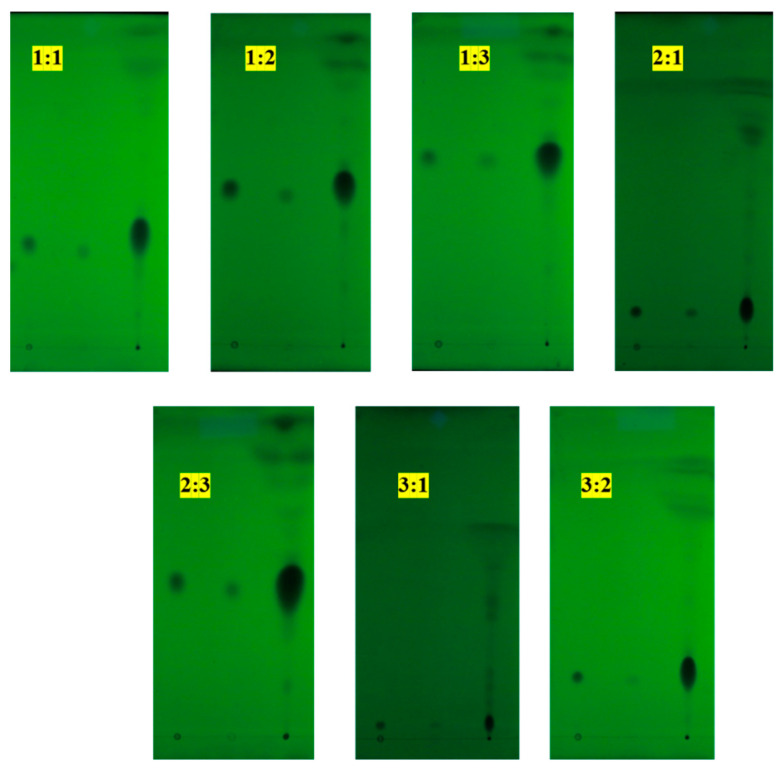
Seven different solvent systems in TLC (the sample points in all TLC plates, from left to right, are standard hydroxyl-α-sanshool and PEE, respectively).

The mobile phase at seven different ratios (*v*/*v*) of petroleum ether (60–90 °C) and ethyl acetate was set in this experiment. The results of thin-layer chromatography (TLC) detection are shown in Figure 1, and the Rf values of the seven different solvent systems are listed (Table 1). According to the results of TLC detection, the seven different solvent systems showed different Rf values in the thin-layer board. First, when the ratios of petroleum ether (60–90 °C):ethyl acetate were 3:1 or 2:1, both of their Rf values were less than 0.20. Moreover, according to Figure 1, the eluting points of standard hydroxyl-α-sanshool and PEE (petroleum ether extract) were really close to the marked points but could not obtain appreciable results of separation. Second, the solvent system of petroleum ether (60–90 °C)–ethyl acetate at a ratio of 1:3 led to an Rf value greater than 0.5, which was not applicable for subsequent elution purification. Third, the petroleum ether (60–90 °C)–ethyl acetate system in different volume ratios of 1:1, 1:2, 2:3, and 3:2 produced a good separation effect in hydroxyl-α-sanshool, whose Rf values ranged between 0.2 and 0.5. Among them, it was found that the separation effect by the component volume ratio of 3:2 was the best. Therefore, petroleum ether (60–90 °C) and ethyl acetate at a 3:2 ratio, with an Rf value of 0.23, were chosen as the eluent. The following are two reasons for choosing the ratio of 3:2 of petroleum ether (60–90 °C) and ethyl acetate: On one hand, when the Rf value is too low (lower than 0.2), it will take a long time to separate the target substance and the target substance may not be separated successfully, which would cause an extreme waste of time and solvent; on the other hand, when the Rf value is more than 0.5, the target substance might easily be eluted out, following the greater polarity impurities, so that the high purity requirements cannot be achieved [42].

### 2.2. Selection of Enriched Method

**Figure 2:**
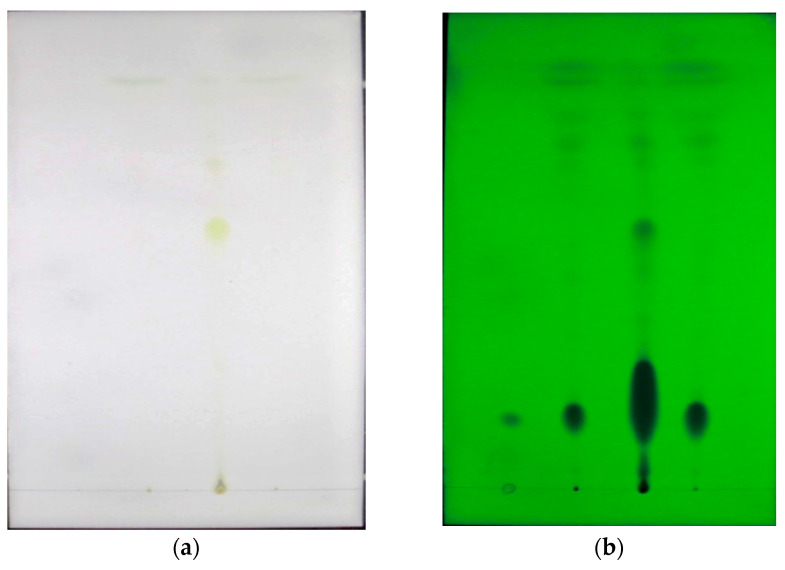
PEE, EEE, and E-PEE TLC (,the sample points in the two TLC plates, from left to right, are standard hydroxyl-α-sanshool, PEE, EEE, and E-PEE, respectively, (**a**) was captured in natural light, (**b**) was taken in UV light).

Figure 2 shows the TLC detection results of the different extracts (PEE, ethyl acetate extract (EEE), and ethyl acetate-petroleum ether extract (E-PEE)) by three enriched methods. Figure 2a was captured in natural light, while Figure 2b was taken in UV light. Comparing Figure 2a with Figure 2b, there are three key points that can be realized. Firstly, the suspected content of hydroxyl-α-sanshool is located in the colorless area under colored compounds on the silica gel TLC plate. Therefore, from this information, it can be inferred that the sample should be collected after the column has been eluted to be colorless. Secondly, the location of the hydroxyl-α-sanshool suspect is in an independent area, which is far from the impurities. Additionally, due to this result, the purification of hydroxyl-α-sanshool from *Z. armatum* using silica gel chromatography might be achieved. Thirdly, it is easy to observe that the EEE keeps far more colored impurities than the PEE and E-PEE. Hence, the EEE might not be suitable for subsequent elution because too many colored impurities would affect the purification of hydroxyl-α-sanshool. In addition, according to the eluting result, the EEE failed to be eluted to colorless even up to 8 h. This result further confirmed that the EEE contained a lot of impurities, and it was not beneficial to separate the hydroxyl-α-sanshool. There were two reasons that might account for this inference. For one thing, the hydroxyl-α-sanshool might have been eluted with colored impurities during the eluting process. For another, the hydroxyl-α-sanshool might also have stayed in the chromatographic column, so this enriching method (EEE) would lead to the consumption of time and eluent. Therefore, the PEE and E-PEE were used in subsequent eluting experiments.

### 2.3. Detection of Hydroxyl-α-Sanshool

**Figure 3:**
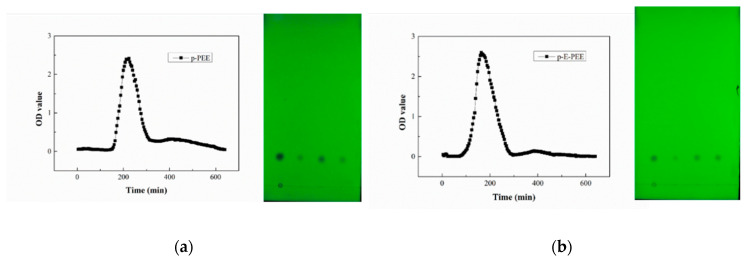
(**a**) UV detection. (**b**) TLC detection (the sample points in the two TLC plates, from left to right, are standard hydroxyl-α-sanshool and the starting point, peak point, and end point of the target fractions, respectively).

The targeted sample was determined by UV and TLC detection. UV and TLC can be used for the qualitative examination of hydroxyl-α-sanshool [42]. These two methods are efficient in detecting targeted substances and useful for the real-time monitoring of the separation process of silica gel chromatography. UV detection is a simple and applied method to target the solvent containing hydroxyl-α-sanshool in the 160 tubes of eluent. The TLC used here was also helpful in further confirming the target substance and tentatively identifying the hydroxyl-α-sanshool in each fraction. All of the collected effluents were detected by UV after proper dilution, and the targeted fractions were preliminarily identified according to the OD value. Then, the starting point, end point, and peak point of target fractions were detected by TLC after concentration by pressure-blowing concentrator. The detected results of UV and TLC are shown in Figure 3. In Figure 3a,b, it is obvious that the three points have the same Rf value with the standard hydroxyl-α-sanshool. Therefore, the eluents of the targeted peaks were combined to get rid of the solvent. Later, the remaining substance appeared to be yellowish and oily. After being refrigerated overnight at −20 °C, the sample presented as a pale-yellow powder, and its state was consistent with the standard hydroxyl-α-sanshool. At a sensory tasting, it presented a strong tingling sensation, i.e., a numbing taste.

### 2.4. Purity and Yield of Hydroxyl-α-Sanshool by Silica Column Chromatography

**Figure 4:**
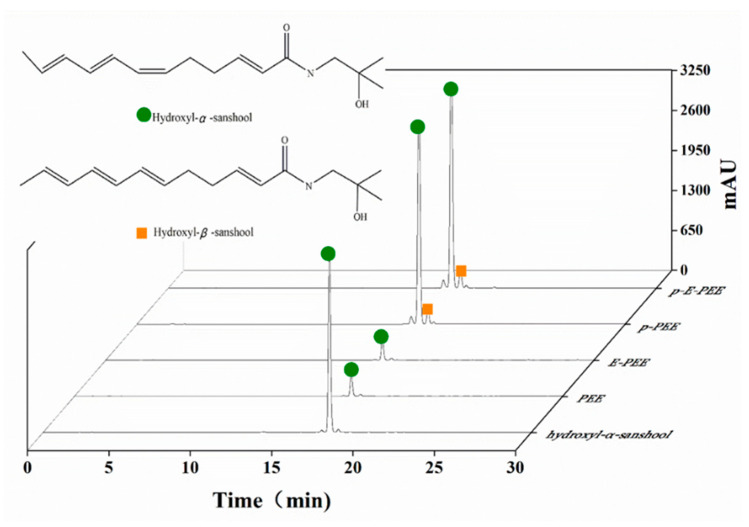
HPLC analysis of hydroxyl-α-sanshool standard and samples.

Silica gel chromatography was investigated for the separation of hydroxyl-α-sanshool from *Z. armatum*. The results of HPLC detection are shown in Figure 4. Figure 4 compares the results of all samples with the standard solution of hydroxyl-α-sanshool; the second peak of all the samples (tR = 17.32 min) is the extract target of this experiment. Then, the purity of all samples (PEE, E-PEE, purified PEE (p-PEE), and purified E-PEE (p-E-PEE)) was calculated based on the results of HPLC detection. The purity results of the four samples are shown in Table 2.

From the calculation results of the external calibration curve method (shown in Table 2), it can be concluded that the hydroxyl-α-sanshool in the p-PEE and p-E-PEE, with purity rates of 93.07% and 98.34%, was obtained, and the yields of the two eluted extraction samples were 7.32% and 12.42%, respectively. Additionally, the recovery rates of hydroxyl-α-sanshool in the p-PEE and p-E-PEE were 107.58% and 121.65%, respectively. Therefore, it could be clearly seen that the purity, yield, and recovery rates of the hydroxyl-α-sanshool obtained by p-E-PEE were higher than those of the hydroxyl-α-sanshool in the p-PEE.

In Table 2, the purity of the PEE and E-PEE was calculated by the methods of the external calibration curve, and the normalization rates were 6.34% and 88.42%, 10.04% and 89.41%, respectively. Compared with these two calculation methods, it was clear that there were significant differences in the results of the purity of the PEE, and the same conclusion could be obtained from the E-PEE. This was because the HPLC detection conditions could only detect sanshools and could not detect other impurities in the PEE and E-PEE. As a result, the peak area of hydroxyl-α-sanshool was relatively large so as to obtain a high purity of PEE and E-PEE. Therefore, the normalization method might not be suitable for calculating the purity of hydroxyl-α-sanshool, which was eluted by the PEE and E-PEE. Hence, compared with the PEE and E-PEE, the purity of hydroxyl-α-sanshool in p-PEE and p-E-PEE increased by 86.73% and 88.30%, respectively. Additionally, according to the calculation results of the external calibration curve method, the purity of hydroxyl-α-sanshool in p-E-PEE could reach 98.34%, which is a higher purity rate than for p-PEE. Overall, compared with p-PEE, the purity, yield, and recovery rate of hydroxyl-α-sanshool in p-E-PEE showed a better separation result, which meant that *Z. armatum* oleoresin (ZAO) extracted by the ethyl acetate first and then extracted by hot petroleum ether (60–90 °C) could obtain a good separated effect in the purification of hydroxyl-α-sanshool.

## 3. Material and Methods

### 3.1. Materials and Chemicals

The samples of *Z. armatum*, harvested and dried in July 2020, were collected from *Hongya* county, Sichuan province, China. The dried *Z. armatum* fruits for this research were full-grained, with complete oil cells.

Ethanol (EtOH), petroleum ether (60–90 °C), and ethyl acetate were purchased from Chengdu Jere Technology Co., Ltd. (Chengdu, China). HPLC grade methyl alcohol and acetonitrile (ACN) were purchased from Thermo Fisher Scientific Inc. (Waltham, MA, USA). Standard hydroxyl-α-sanshool was purchased from Chengdu RefMedic Biotech Co., Ltd. (Chengdu, China, purity ≥ 98.00%). Silica gel (100–200 mesh) and thin-layer chromatography (TLC) plates (silica gel GF254, 100 × 200 mm) were obtained from Qingdao Haiyang Chemical Co., Ltd. (Qingdao, China). Ultrapure water was prepared using a Millipore purifier (Millipore, Burlington, MA, USA).

### 3.2. Extraction of Hydroxyl-α-Sanshool

The extraction of hydroxyl-α-sanshool was performed according to Bhatt et al. [43], with some modifications. First, the raw material was crushed by a universal high-speed pulverizer (FW-100, Beijing Zhongxing Weiye Instrument Co., Ltd., Beijing, China) and passed through a 40-mesh sieve. Next, the *Z. armatum* powder was mixed with 70% EtOH at a ratio of 1:10 (*w*/*v*) to extract hydroxyl-α-sanshool at room temperature for 2 h, and then, the mixture was extracted with ultrasound (KH-50B, Kunshan Ultrasonic Instrument Co., Ltd., Kunshan, China) at 30 °C for 15 min. After being vacuum filtered by a Buchner funnel, the filtrate was concentrated by a rotary vacuum evaporator at 53 °C to obtain a pasty residue, i.e., *Z. armatum* oleoresin (ZAO).

### 3.3. Selection of Solvent System

Non-aqueous two-phase solvent systems based on petroleum ether (60–90 °C) and ethyl acetate, with seven kinds of different ratios, were investigated to select a proper ratio for subsequent elution using thin-layer chromatography (TLC) detection. The method of TLC was carried out according to Kashiwada et al. [14], with some modifications. The PEE was placed on silica gel TLC plates. The mobile phases at seven different ratios (*v*/*v*) of petroleum ether (60–90 °C) and ethyl acetate were 1:1, 1:2, 1:3, 2:1, 2:3, 3:1, and 3:2, respectively. After chromatographic separation, the TLC plates were exposed to ultraviolet (UV) light (WD-9403C, Beijing Liuyi Instrument Co., Ltd., Beijing, China), and the Rf values were calculated. An appropriate mixed ratio (Rf values ranged from 0.2 to 0.3) of petroleum ether (60–90 °C) and ethyl acetate was selected as the solvent system for silica gel chromatographic elution and subsequent TLC detection.

### 3.4. Enrichment of Hydroxyl-α-Sanshool

In this experiment, two kinds of organic solvents for the three methods were severally used to enrich hydroxyl-α-sanshool, i.e., petroleum ether (60–90 °C) and ethyl acetate. The enrichment of hydroxyl-α-sanshool was carried out according to Li et al. [44], and some modifications were made based on previous research in our laboratory [45]. The ZAO was dissolved in ultrapure water at a ratio of 1:10 (*w*/*v*) and then extracted with hot petroleum ether (60–90 °C) in the same volume as the ultrapure water. After stratification by a separatory funnel, the extracted organic layers were freed from the solution and evaporated in a rotary vacuum evaporator to get the petroleum ether extract (PEE). The same experimental steps were followed to obtain the ethyl acetate extract (EEE) when ethyl acetate was used instead of petroleum ether (60–90 °C). In addition, the third method to enrich hydroxyl-α-sanshool was to extract ZAO by ethyl acetate first and then by hot petroleum ether (60–90 °C), which could obtain the ethyl acetate–petroleum ether extract (E-PEE). Among these three enrichment methods (PEE, EEE, and E-PEE) of hydroxyl-α-sanshool from ZAO, the proper extracts were chosen by TLC detection, which would be prepared for the subsequent elution and purification of hydroxyl-α-sanshool using silica gel column chromatography.

### 3.5. Silica Gel Chromatography

Around 2.00 g of extract (PEE, EEE, or E-PEE) was stirred evenly with 2.50 g silica gel and then loaded on the top of silica gel in a silica gel column (40 cm × 1.6 cm). The column was eluted, employing the solvent mixture (about 1200 mL) of petroleum ether (60–90 °C) and ethyl acetate, the ratio of which was determined by the results of Part 3.3. Chromatography was performed with a two-phase eluent at a flow rate of about 1.5 mL/min. Moreover, the effluent was collected in a fraction size of 4 mL after the silica gel was eluted to colorless, and a total of 160 tubes were collected. Tentative identification of the aimed substance was carried out using UV (UV-3100N, Jiangsu Ronghua Instrument Manufacturing Co., Ltd., Suzhou, China) and TLC. Among them, the separated and selected fractions (containing hydroxyl-α-sanshool) of the PEE and E-PEE using silica gel chromatography were purified PEE (p-PEE) and purified E-PEE (p-E-PEE).

### 3.6. UV Detection

All collected fractions were diluted using the mixed solvent of petroleum ether (60–90 °C) and ethyl acetate by an appropriate multiple and then detected by UV in turn. Combined with the results of TLC detection, the fractions containing hydroxyl-α-sanshool were pooled and concentrated by rotary evaporation and then dried by a pressure-blowing concentrator (MTN-2800D, Tianjin Autoscience Instrument Co., Ltd., Tianjin, China).

### 3.7. TLC Detection

Thin-layer chromatography (TLC) is a chromatographic separation technology that uses the support coated on the support plate as the stationary phase and the appropriate solvent as the mobile phase to separate, identify, and quantify the mixed samples. In this work, TLC was applied to detect the purification efficiency of hydroxyl-α-sanshool.

According to the results of UV detection, the fractions of the starting point, end point, and peak point of the eluent that contained the target substance segment were selected and concentrated by a pressure-blowing concentrator. The concentrated solution, along with the standard solution, was loaded to the marked points above 1.5 cm from the bottom of the silica gel plate. After chromatographic separation, the sample spots were visualized by UV light, with standard hydroxyl-α-sanshool for identification, and the testing results were recorded by a digital camera (70D, Canon, Tokyo, Japan).

### 3.8. HPLC Analysis

The enriched extracts (PEE and E-PEE) and selected fractions (p-PEE and p-E-PEE) were analyzed by HPLC. The chromatographic conditions were maintained according to Ke et al. [1]. The data of hydroxyl-α-sanshool were obtained with an Agilent 1260 series HPLC-DAD system (Agilent, Santa Clara, CA, USA). The chromatographic separation was performed on an Agilent Eclipse XDB-C18 column (4.6 × 150 mm, 5 μm), and the temperature was maintained at 35 °C. The mobile phase consisted of acetonitrile (A) and deionized water (B), with a linear gradient program as follows: 0 min, 35% A; 5–10 min, 35–40% A; 25–50 min, 45–65% A; 55–60 min, 90–35% A; and the flow rate at 0.8 mL/min. The injection volume was 10 μL, and the detector wavelength was set at 268 nm. The gradient concentration of standard hydroxyl-α-sanshool was: 0, 0.1, 0.2, 0.3, 0.4, 0.5, 0.6 mg/mL.

### 3.9. Purity Calculation

According to the results of HPLC, the methods of normalization and the external calibration curve were used to calculate the purity of the purified hydroxyl-α-sanshool.

Normalization method: The ratio of the peak area of hydroxyl-α-sanshool to the sum of all peak areas, based on the results of HPLC detection, is the purity of the hydroxyl-α-sanshool.

External calibration curve method: The concentrations of hydroxyl-α-sanshool in PEE, E-PEE, p-PEE, and p-E-PEE are calculated based on the standard curve line of hydroxyl-α-sanshool, according to the results of HPLC detection. Then, the ratio of the calculated concentration of hydroxyl-α-sanshool, which is calculated by the method of the external calibration curve to the concentration of the configuration solution (0.5 mg/mL), is the purity of the hydroxyl-α-sanshool. The concentration of the configuration solution is determined by a certain weight extract (PEE, E-PEE, p-PEE, and p-E-PEE) dissolved in an acetonitrile–water solution.

### 3.10. Yield and Recovery Rate

Silica gel chromatography was investigated for separating the hydroxyl-α-sanshool from *Z. armatum*. Then, the yield and recovery rate of purified hydroxyl-α-sanshool were calculated [46]. The yield of hydroxyl-α-sanshool is calculated using Equation (1).

(1) $$Y\;(\%)=\frac{W_{S}}{W_{T}}\;\text{×}\;100\;$$

where *W_S_* and *W_T_* are the weight of p-PEE or p-E-PEE and PEE or E-PEE, respectively.

The recovery rate of purified hydroxyl-α-sanshool is calculated according to Equation (2).

(2) $$R\;(\%)=\frac{W_{S}\times P_{S}}{W_{T}\times P_{T}}\times 100$$

where *W_S_* and *W_T_* are the weight of the p-PEE or p-E-PEE and PEE or E-PEE, respectively; *P_S_* and *P_T_* are the purity of hydroxyl-α-sanshool in p-PEE or p-E-PEE and PEE or E-PEE, respectively.

### 3.11. Statistical Analyses

Multiple parallel samples were tested, and the data are expressed as mean ± standard deviation. Data were processed and plotted using SPSS 20 and Origin 9 (Origin Lab, Northampton, MA, USA). Duncan’s multiple analysis of variance was performed using ANOVA (*p* < 0.05).

## 4. Conclusions

A sample and rapid method for the separation and purification of hydroxyl-α-sanshool from *Z. armatum* has been established. A hydroxyl-α-sanshool extract was subjected to silica gel column chromatography and was eluted using a binary solvent mixture of petroleum ether (60–90 °C) and ethyl acetate (3:2, *v*/*v*). The eluent was collected in a fraction size of 4 mL, and tentative identification was carried out using TLC and UV. According to the results, the purity, recovery rate, and yield of hydroxyl-α-sanshool in p-E-PEE were higher than those in p-PEE. Therefore, p-E-PEE was chosen as the eluted target to purify the hydroxyl-α-sanshool from *Z. armatum*. The fractions containing hydroxyl-α-sanshool in p-E-PEE were pooled and dried by rotary evaporation and hydroxyl-α-sanshool was obtained with a purity of 98.34%; the yield and recovery rates were 12.42% and 121.65%, respectively. Additionally, compared with E-PEE, the purity of hydroxyl-α-sanshool in the purification of p-E-PEE increased by 88.30%. The purity of hydroxyl-α-sanshool separation by silica gel column chromatography was enough to research its physiological and food processing activities in *Z. armatum.*

## Figures and Tables

**Table 1:** Rf of hydroxyl-α-sanshool in different solvent systems.

Petroleum Ether/Ethyl Acetate (*v*/*v*)	Rf
1:1	0.32
1:2	0.49
1:3	0.58
2:1	0.13
2:3	0.47
3:1	0.07
3:2	0.23

**Table 2:** The purity, yield, and recovery yield of all samples.

Samples	External Standard Method (%)				Normalization Method (%)			
	Purity	RSD	Yield	Recovery Rate	Purity	RSD	Yield	Recovery Rate
PEE	6.34	0.26			88.42	0.40		
E-PEE	10.04	0.69			89.41	0.47		
p-PEE	93.07	1.77	7.32	107.58	89.30	0.46	7.32	103.22
p-E-PEE	98.34	0.46	12.42	121.65	90.65	0.55	12.42	112.14

## Data Availability

Not applicable.
